# *Calligonum polygonoides* L. Shrubs Provide Species-Specific Facilitation for the Understory Plants in Coastal Ecosystem

**DOI:** 10.3390/biology9080232

**Published:** 2020-08-17

**Authors:** Ahmed M. Abd-ElGawad, Younes M. Rashad, Ahmed M. Abdel-Azeem, Sami A. Al-Barati, Abdulaziz M. Assaeed, Amr M. Mowafy

**Affiliations:** 1 Plant Production Department, College of Food & Agriculture Sciences, King Saud University, P.O. Box 2460, Riyadh 11451, Saudi Arabia; assaeed@ksu.edu.sa; 2 Department of Botany, Faculty of Science, Mansoura University, Mansoura 35516, Egypt; ammr79@mans.edu.eg; 3 Plant Protection and Biomolecular Diagnosis Department, Arid Lands Cultivation Research Institute, City of Scientific Research and Technological Applications (SRTA-City), New Borg El-Arab City 21934, Egypt; younesrashad@yahoo.com; 4 Department of Botany, Faculty of Science, Suez Canal University, Ismailia 41522, Egypt; ahmed_abdelazeem@science.suez.edu.eg; 5 Biology Department, Faculty of Science, Sana’a University, Sana’a 15542, Yemen; aboabdalrahmansami75@gmail.com

**Keywords:** nurse plant, mycorrhizae, arid habitat, proline, species coexistence, positive interactions, allelopathy

## Abstract

Plant facilitation has a pivotal role in regulating species coexistence, particularly under arid environments. The present study aimed to evaluate the facilitative effect of *Calligonum polygonoides* L. on its understory plants in coastal habitat. Forty *Calligonum* shrubs were investigated and the environmental data (soil temperature, moisture, pH, salinity, carbon and nitrogen content, and light intensity), vegetation composition, and diversity of associated species were recorded under- and outside canopies. Eight of the most frequent understory species were selected for evaluating their response to the facilitative effect of *C. polygonoides*. Bioactive ingredients of *Calligonum* roots were analyzed using gas chromatography-mass spectrometry (GC-MS), and mycorrhizal biodiversity in their rhizosphere soil was also assessed. The effect of *Calligonum* on understory plants ranged between facilitation and inhibition in an age-dependent manner. Old shrubs facilitated 18 and inhibited 18 associated species, while young shrubs facilitated 13 and inhibited 9 species. *Calligonum* ameliorated solar radiation and high-temperature stresses for the under canopy plants. Moreover, soil moisture was increased by 509.52% and 85.71%, while salinity was reduced by 47.62% and 23.81% under old and young shrubs, respectively. Soil contents of C and N were increased under canopy. This change in the microenvironment led to photosynthetic pigments induction in the majority of understory species. However, anthocyanin, proline contents, and antioxidant enzyme activities were reduced in plants under canopy. Thirteen mycorrhizal fungal species were identified in the rhizospheric soil of *Calligonum* with the predominance of *Funneliformis mosseae*. Thirty-one compounds were identified in *Calligonum* root extract in which pyrogallol and palmitic acid, which have antimicrobial and allelopathic activities, were the major components. The obtained results demonstrated that facilitation provided by *Calligonum* is mediated with multiple mechanisms and included a set of interrelated scenarios that took place in a species-specific manner.

## 1. Introduction

Arid regions represent about 40% of the global land area, in which environmental conditions are known to exert a strong influence on organisms to the extent of hindering their growth and development [1]. To overcome desertification in these habitats, shrubs that are able to survive in such conditions can be used to restore vegetation as they create more mesic spots for other understory plants. Such plants are termed as nurse plants and the phenomenon that is represented by plant-plant positive interactions is called plant facilitation [2]. Nurse plants are characterized by their ability to sustain life, growth, and physiology of neighboring plants. The canopy of the nurse plant ameliorates the effect of light and temperature on the understory species, and inconsequence modulates the physiological response such as photosynthetic rate, respiration, and enzyme activity [2,3]. In contrast, individuals in the same community might compete with each other for nutrients, light, space, and water uptake. The concept of plant facilitation was not common until recently when it has been re-evaluated particularly in extreme habitats [4]. Whether through facilitation or competition, these interactions shape the plant community structure, particularly under severe conditions in which the interactions between plants are shifted toward facilitation [5,6] as in deserts and salt marshes as predicted by the stress gradient hypothesis (SGH). Moreover, this idea was followed by other models proposing the collapse of facilitation in extremely harsh conditions [7]. The main facilitation benefits given by the overstory shrubs are through alleviation of the abiotic stresses, and providing more favorable conditions for the understory species such as buffered temperatures, increased shading, soil moisture, nutrition, protection from herbivores, and enhancement of beneficial soil microorganisms making the conditions more favorable to the understory plants [4,8]. Additionally, the nurse plant exudates a vast number of chemicals for roots. These chemicals comprise a mixture of organic acids, sugars, enzymes, amino acids, nucleotides, and other bioactive compounds such as alkaloids, phenolics, glycosides, and volatile organic compounds [9,10,11]. These exudates have either stimulatory or inhibitory (allelopathic) activity for the understory plant species as well as the microflora [2].

The mutualistic relationships established between arbuscular mycorrhizal fungi (Glomeromycotina) and terrestrial plants play key roles in the establishment, diversification, productivity, and sustainability of different natural ecosystems [12]. Furthermore, arbuscular mycorrhizal fungi are considered as a crucial factor in the afforestation programs [13] as they enhance soil texture and fertility [14]. This symbiotic relationship may directly or indirectly benefit the understory plants as well as the nurse plant through the formation of complex common mycorrhizal networks (CMNs) serving as channels by which the plants could communicate. These CMNs provide the interconnected plants with important benefits concerning water and nutrients transport, and signal exchange, affecting their plant vigor, survival, and competition [15]. In the CMN-interlinked plant community, the weak plant individuals can benefit from nutrients supply and other advantages from the stronger nurse plant(s). In other words, the nurse plant reduces competition between the other interconnected weak plants allowing facilitation [2]. However, the impact of CMNs on the interconnected plants is highly species-dependent [16].

*Calligonum polygonoides* L. inhabits the North African desert and sandy deserts of the Middle East. It is a common shrub in the Mediterranean coastal zone of Egypt, where it dominates the sandy habitats. It is a much-branched shrub capable of building sand mounds (nebka). Moreover, *C. polygonoides* has ecological importance owing to its ability to stabilize the mobile sand dunes, prevent erosion, and increase the organic matter content in the soil [17]. As a nurse plant, *C. polygonoides* has been proved to be a potential soil ameliorating factor, as well as a facilitative herbaceous plant in India [18] and Iran [19]. The Mediterranean coastal belt habitat in Egypt is highly sensitive due to its vulnerability to inundation and saltwater intrusion as a result of sea-level rise. The plant communities of this habitat are suffering from many influences including urbanization, agriculture expansion, pollution, touristic pressures, industrialization, over-collecting, overgrazing, military activities as well as the invasion by alien species [20]. In degraded habitats with harsh environmental conditions, the nurse plants play a vital role in the ecological restoration, where the facilitation process becomes essential for survival, growth, and performance of the nearby plants, as well as improvement of diversity and community dynamics [2,6].

The present study is aimed to (1) determine the effect of *C. polygonoides* shrubs on the understory plant species, (2) evaluate the effects of *C. polygonoides* on the microhabitat under canopy, (3) assess the physiological response of the most frequent understory species to the presence of *C. polygonoides*, (4) investigate the mycorrhizal diversity in the rhizospheric soil of *C. polygonoides*, and (5) analyze the chemical composition of the *C. polygonoides* root extract.

## 2. Materials and Methods

### 2.1. Field Study Area

The study was performed along the Mediterranean coastal belt of the Nile delta, Northern Egypt. The studied location area extended from Damietta (31°26′39.6″ N 31°37′18.9″ E) to Baltim (31°35′25.4″ N 31°02′06″ E), with ≈50 km long. Usually, the coastal regions suffer from saltwater intrusion which increases the salinity level. In addition, this zone in Egypt is more sensitive due to sea level rise as well as climate change. The climate of the northern coast of Egypt is mainly affected by the Mediterranean Sea. The metrological data from 1979 to 2010 revealed that annual average air temperature ranged from 15.2 to 25.9 °C, and the annual average total precipitation was up to 200 mm [21].

### 2.2. Study Species

**Figure 1:**
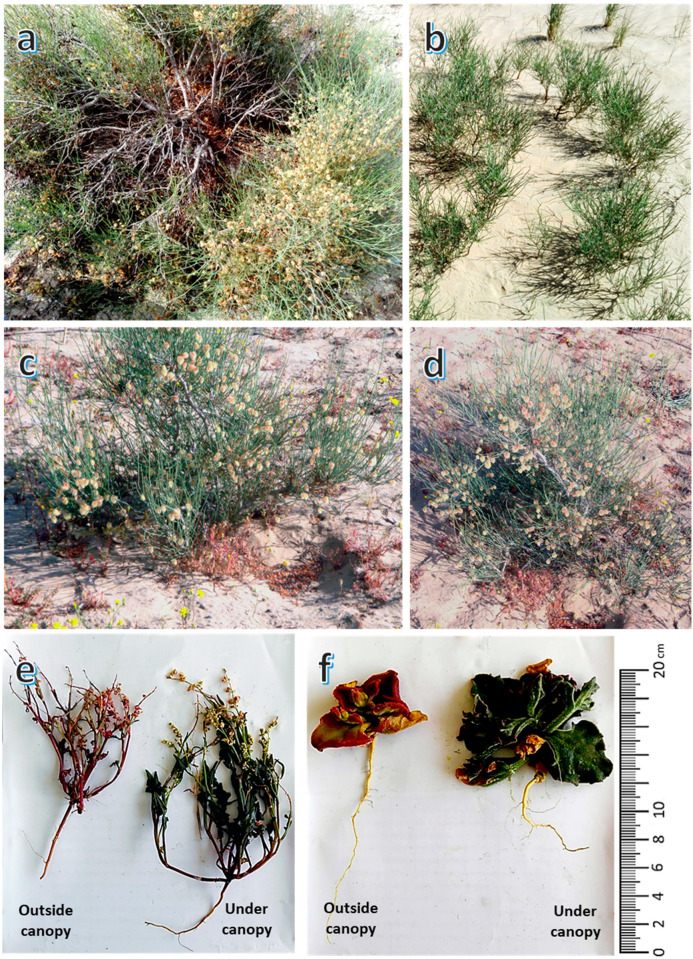
*Calligonum polygonoides* L. shrub and two selected understory species. (**a**) overview of old shrub, (**b**) regenerated vegetative branches, (**c**,**d**) young shrub, (**e**) *Rumex pictus* L. under, and outside the canopy of *C. polygonoides*, and (**f**) *Mesembryanthemum crystallinum* L.

*Calligonum polygonoides* is a common shrub in Egypt and it is considered to be a dominant perennial plant occupying sand soil under water scarcity making it suitable to combat desertification. It grows up to two meters tall and 5 m in diameter on mobile sand dunes, stabilized dunes, and sand sheets (Figure 1). The adult plants have a lignified stem that carries vegetative and generative branches. The vegetative branches become lignified, brown and shiny with age forming the skeleton of the plant. However, the generative branches develop from the accessory buds of the vegetative branches and never lignified, but these generative branches are shed at the beginning of summer i.e., under canopy at the end of the growing season [22]. The taproot of *C. polygonoides* extends to a depth of more than 1.5 m, while the superficial roots horizontally extend by 10–20 m. In addition, *C. polygonoides* builds up ‘nebka’, which is a sand dune that forms around vegetation and is able to rearrange its canopy above the nebka.

### 2.3. Field Sampling and Vegetation Survey

To examine the effects of *C. polygonoides* on the understory plant species, a total of 40 shrubs were surveyed randomly within 40 stands from January to May 2017. These shrubs were regarded as old (with obvious lignification) and young shrubs (with green branches). The canopy width, height, and length were measured for every shrub. In each instance, three quadrats (0.5 m × 0.5 m) were placed under the canopy at 50 cm from the trunk. Within the quadrat, the density of each plant species was measured. Coupled with each shrub, similar quadrats were performed in the gaps outside the canopy (at least 1 m away from the canopy), at which the plant species density was also measured. The frequency percentage of each species within all studied quadrats, of old and young shrubs, was calculated. The taxonomic nomenclature, identification, and chorotype of plant species were assessed according to Boulos [23]. However, life forms were identified according to the scheme of Raunkiaer [24].

### 2.4. Environmental Measurements and Soil Analysis

To evaluate the effect of *C. polygonoides* shrubs on the environmental conditions of the microhabitat, soil moisture, temperature, contents of organic carbon (OC) and total nitrogen (TN), salinity, pH, and available photosynthetic photon flux density (PPFD) under- and outside-canopy were determined. For each quadrat, soil temperature was measured at ≈15 cm depth below the ground surface from 10:00 to 12:00 AM along clear days using soil thermometer (Spectrum, Technologies, Inc., Aurora, IL, USA). Photosynthetic photon flux density (PPFD, λ = 400–700 nm) was measured by a digital lux meter (Model: LX-101, Lutron Electronic, Taipei, Taiwan). The probe was placed 5 cm above the soil surface in a north-south direction. The data of soil moisture, temperature, and PPFD were taken every week, starting from January to May 2017. Therefore, 7650 readings were taken during all the fieldwork 21 weeks × 40 stands × 3 locations (outside, under canopy of old, and young shrubs) × 3 replications.

In each stand, three soil samples (15–20 cm depth) were collected under and outside canopy, packed in polyethylene bags, and brought to the laboratory. The collected samples were then air-dried at room temperature (25 °C ± 2), sieved through 2-mm sieve to remove stones and plant debris, and packed in bags until further physical and chemical analyses. Soil pH and electrical conductivity (EC) were measured in water suspension (1:2.5) as described by Jackson [25]. To estimate TN in soil, one gram of air-dried homogenous soil sample was first digested by using H_2_SO_4_ for 8 h in the presence of 0.5 g catalyst (K_2_SO_4_, CuSO_4_.5H_2_O, and SeO_2_) then the micro-Kjeldahl method was used to estimate total nitrogen as ammonia [25]. The OC content was estimated according to the modified Walkley–Black method [26].

### 2.5. Plant Analyses

According to the data of the vegetation analysis, eight of the most frequent understory plant species within the quadrats were selected. These species are *Bromus diandrus*, *Cakile maritima*, *Erodium laciniatum*, *Launaea mucronata*, *Mesembryanthemum crystallinum*, *M. nodiflorum*, *Rumex pictus*, and *Senecio glaucus*. To evaluate the effects of *C. polygonoides* shrub on these understory species, dry weight, contents of photosynthetic pigments, total anthocyanin, and proline, as well as the activities of some antioxidant enzymes were estimated in the leaves of these understory plants, under- and outside the canopies.

#### 2.5.1. Determination of Dry Weight

Twenty individuals of the selected plants were collected under canopy and outside canopy, packed in polyethylene bags, and transferred to the laboratory. The plant samples were dried in an oven at 65 °C until reaching constant weights.

#### 2.5.2. Estimation of Photosynthetic Pigments

A known fresh weight (0.1 g) of plant leaves was extracted using 80% chilled acetone to determine chorophyll and carotenoids pigments. Chlorophyll pigments were determined according to Arnon [27], while carotenoids were estimated according to Myers and Kratz [28].

#### 2.5.3. Estimation of Total Anthocyanin Content

To a known weight of the plant leaves (0.1 g), 4 mL of chilled acidic methanol (HCl 0.1% *v*/*v*) was added. After maceration and centrifugation at 6000 rpm at 4 °C for 20 min, the spectrum from 300 to 700 nm was monitored using a spectrophotometer (Jenway 7315 UV-VIS, Burlington, VT, USA). Anthocyanin content was calculated using the following formula:

$$Anthocynin\;content=\;Absorbance_{530}-Absorbance_{653}$$

The values obtained were normalized to cyaniding 3-glucoside (Mol. Mass = 449.2 and ε = 26.9 mM^−1^ cm^−1^) concentrations of mg g^−1^ fresh weight [29].

#### 2.5.4. Estimation of Proline Content

A known weight of the plant leaves (0.1 g) was immersed in 2 mL of 3% sulfosalicylic acid. After maceration and centrifugation at 6000 rpm at 4 °C for 20 min, 2 mL of phosphoric acid and 2 mL of ninhydrin reagent were added and the mixture was heated together in a water bath at 100 °C for 1 h and then 4 mL of toluene were added to the mixture to extract the developed color, and then the absorbance was measured at 520 nm using a spectrophotometer (Jenway 7315 UV-VIS, Colombia, MD, USA) [30].

#### 2.5.5. Assays of Antioxidant Enzymes

A known fresh weight of plant leaves (0.1 g) was immersed in liquid-N and ground in 50 mM potassium phosphate buffer (KPB) pH 7. After centrifugation at 6000 rpm at 4 °C for 20 min, the supernatant was collected and kept at −20 °C until use.

For catalase assay (CAT; EC 1.11.1.6), an assay mixture of 500 µL containing 50 mM KPB pH 7 and 10 mM H_2_O_2_ (ε = 0.036 mM^−1^ cm^−1^). The absorbance was measured at 240 nm for 2 min using the kinetic mode of a spectrophotometer (Jenway 7315 UV-VIS, USA). One unit of enzyme activity is the decomposition of 1 µM H_2_O_2_ per min mL^−1^. Peroxidase activity (POD; EC 1.11.1.7) was assayed in 500 µL reaction mixture containing 50 mM KPB pH 7, 50 mM pyrogallol, and 0.03% H_2_O_2_. The increase in absorbance due to the production of purpurogallin (ε = 2.47 mM^−1^ cm^−1^) was continuously measured at 420 nm for two min. Glutathione reductases (GR; EC 1.6.4.2) activity was assayed by using biodiagnostic kit. A mixture (500 µL) containing 80 mM KPB pH 7.5, 0.8 mM EDTA, 4 mM oxidized glutathione (GSSG) and 0.16 mM NADPH was used. The decrease in NADPH (ε = 6.27 mM^−1^ cm^−1^) concentration at 340 nm was measured over two minutes [31].

Superoxide dismutase activity (SOD, EC 1.15.1.1) was assessed via the inhibition of nitro blue tetrazolium chloride (NBT) reduction using biodiagnostic kit in which an assay mixture of 500 µL contained 40 mM KPB pH 8.5, 0.1 mM NBT, 0.1 mM NADH and 0.01 mM Phenazine methosulphate (PMS). The absorbance was measured for both samples and blank for 2 min at 560 nm. The amount of enzyme required for 50% inhibition of NBT was considered as one unit of SOD activity [32].

### 2.6. Mycorrhizal Biodiversity Analysis

To study the species and diversity of mycorrhizal fungi, rhizospheric soil samples, under the canopy of *C. polygonoides* shrub, were collected from 12 stands. The stands were selected randomly, at least one km far from each other. The mycorrhizal spores were extracted using the wet sieving and decanting method described by Gerdemann and Nicolson [33] and density gradient centrifugation [34], and then counted using a dissecting microscope. For identification, mycorrhizal spores were mounted on glass slides in polyvinyl-lactophenol-glycerol under a dissecting microscope. Morphological characteristics were described according to Schenck and Perez [35] and the species description webpage (https://invam.wvu.edu/).

Spore density, relative abundance, species richness, and frequency of mycorrhizal fungi were estimated as follows: spore density (SD) = number of mycorrhizal spores in 50 g of soil; species richness (SR) = number of fungal taxa found in 50 g of soil; relative abundance (RA) = (number of spores of a species or genus/total spores) × 100, and frequency (F) = (number of samples in which the species or genus was observed/total samples) × 100.

Mycorrhizal colonization levels were evaluated in roots of the eight selected plant species grown under and outside canopy of *C. polygonoides*. Plant roots were cut into 1 cm segments, stained with trypan blue [36], and forty root segments from each plant species were examined using a compound microscope (×400), and the colonization levels were estimated according to Trouvelot et al. [37].

### 2.7. Chemical Composition Analysis of C. polygonoides Roots

Roots of *C. polygonoides* with healthy appearance were collected from different stands along the Mediterranean coast of Egypt. For extraction, 80 g of air-dried root sample was ground into a fine powder, added to 250 mL 80% (*v*/*v*) methanol in a dark bottle, and shaken at 150 rpm on a rotary shaker for 72 h at room temperature (25 °C ± 2). The crude extract was firstly filtered through Whatman filter paper No. 1, then centrifuged at 4000 rpm for 60 min. The supernatant was collected in a clean dark-glass-bottle, and freeze-dried using vacuum lyophilizer (FZ-6, Labconco Co., Kansas City, MO, USA). The residue was re-dissolved in 10 mL of methanol (HPLC grade) and re-filtered using a micro-filter (0.45 µm pores).

The root extract of *C. polygonoides* was analyzed using the GC-MS-QP 2010 system (Shimadzu, Kyoto, Japan) to investigate its chemical composition. The sample was injected at a rate of 1 mL min^−1^ via DB-5 column (60 m × 0.25 mm, 0.25 μm thick) using helium as a carrier at 250 °C. The oven temperature was 50 °C using the split mode of injection at 50:1. The ion source temperature was 230 °C, but the interface temperature was 250 °C, at an ionization voltage of 70 eV. The retention times, as well as mass spectra of the constituents, were used to identify the chemical components of the extract using the NIST11 mass spectral database (Gaithersburg, MD, USA).

### 2.8. Data Analysis

To measure the effect of *C. polygonoides* shrubs on the understory plant species, the relative interaction index (RII) was calculated. Based on the data of vegetation analysis (relative densities), and according to Armas et al. [38], RII of each associated plant species was calculated according to the following equation:

$$RII=\left(RD_{Under\;canopy}-RD_{Ouside\;canopy}\right)/(RD_{Under\;canopy}+RD_{Ouside\;canopy)}$$

where RD is the relative density of the associated species. The RII determines the intensity of change in the presence of a shrub (under canopy) compared to gaps (outside canopy) and ranges from −1 (indicating negative effects) to +1 (facilitation). In addition, RIIs for the eight selected species based on their biomass (dry weight) were also calculated.

The plant species diversity, as well as mycorrhizal species diversity, were determined by calculating species richness (Simpson index) and species evenness (Shannon-evenness) according to the following equations:

$$Simpson\;index\;\left(S\right)\;=\frac{\sum_{i}\left[n_{i}\times \left(n_{i}-1\right)\right]}{\left[N\times \left(N-1\right)\right]}$$

$$Shannon-Evenness\;index\;\left(H\right)=\overset{s}{\underset{i=1}{\sum }}P_{i}\ln(P_{i})$$

$$Shannon-Wiener\;index\;\left(E\right)=\frac{H}{ln_{s}}$$

where *Pi = ni/N* = proportional abundance of species, *i* in a habitat made up of *s* species, *n_i_* = the number of quadrats containing species *i* and *N* = S *n_i_*.

Soil data (temperature, PPFD, and chemical analyses), as well as plant biochemical analyses (photosynthetic pigments, total anthocyanin, proline, and antioxidant enzymes), were subjected to one-way ANOVA followed by Duncan’s test with a probability level of 0.05 using CoStat 6.311 (CoHort Software, Monterey, CA, USA). The data were tested for the normality test and homogeneity of variance with no violation before running ANOVA. The data of shrubs measurements were subjected to two-tailed *t*-test at the probability level of 0.05 using XLSTAT 2018 (Addinsoft, New York, NY, USA).

## 3. Results

### 3.1. Vegetation Analysis

A significant variation in shrub canopy width, length, and height was observed between old and young *C. polygonoides* (Figure S1). The vegetation analysis of the associated species with *C. polygonoides* shrubs revealed the presence of 40 plant species (15 perennials and 25 annuals), belonging to 16 families (Table S1). The most represented families were Poaceae (22.5%), Asteraceae (20.0%), and Chenopodiaceae (15.0%).

**Figure 2:**
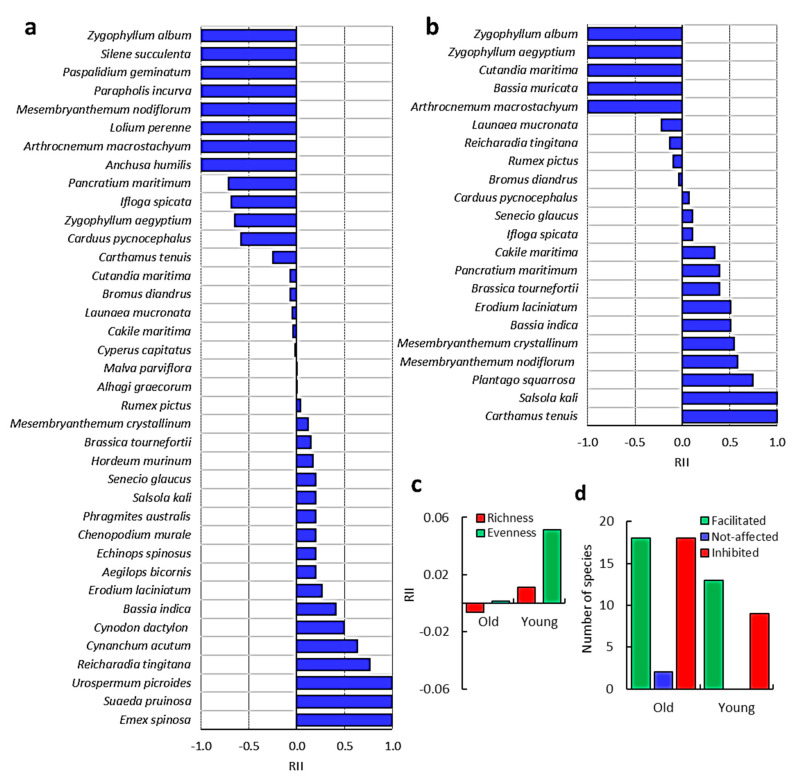
Interference of *C. polygonoides* shrubs with the associated species. (**a**) Relative interaction index (RII) of the associated species with old shrubs. (**b**) RII of the associated species with young shrubs. (**c**) RII of *C. polygonoides* shrubs on the associated species richness and evenness. (**d**) Number of facilitated, inhibited or not-affected associated species.

**Figure 3:**
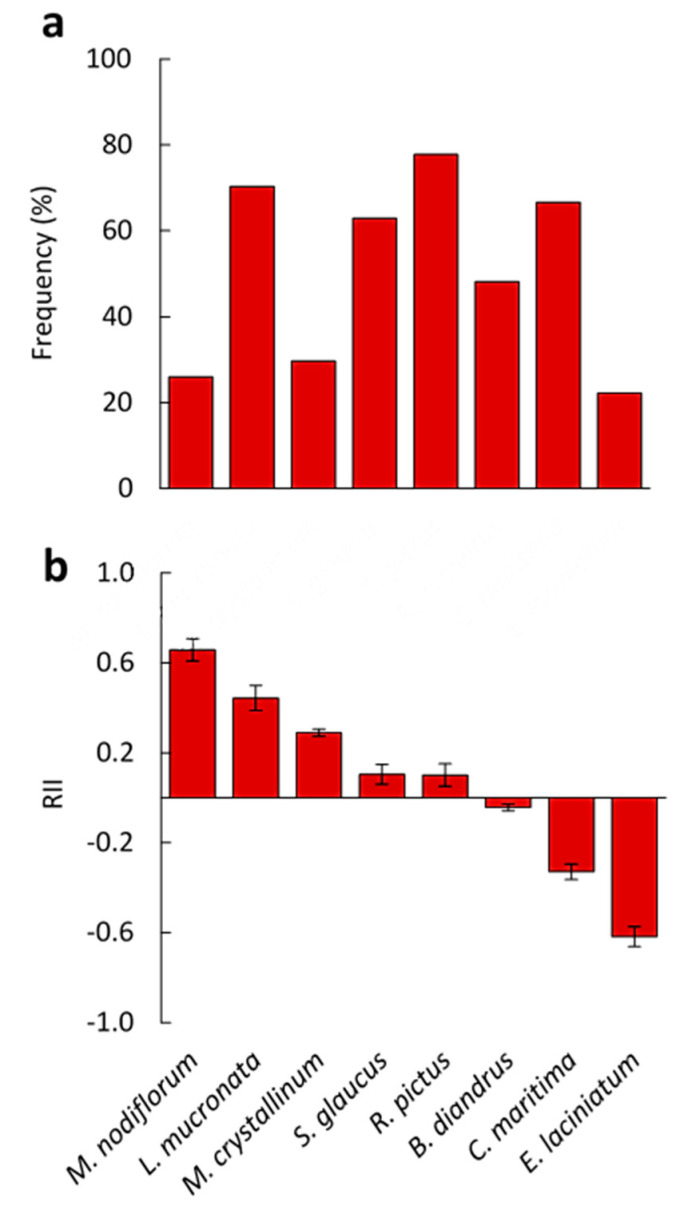
Interaction of *C. polygonoides* shrubs (old and young shrubs) with the eight selected associated species. (**a**) Frequency percentage of the eight selected species (highest one). (**b**) Relative interaction index (RII) values based on biomass (dry weight, *n* = 20).

The analysis of RII, based on the relative densities of the associated species with old and young *C. polygonoides* shrubs revealed varied effects ranged from facilitation to inhibition (Figure 2). The old shrubs provided facilitation for 18 and inhibited 18 associated plant species (Figure 2a), while the young shrubs provided facilitation to 13 and inhibited 9 plant species (Figure 2b). Based on the frequency of the associated plant species, eight species attained frequency higher than 22.22%. These species are *B. diandrus*, *C. maritima*, *E. laciniatum*, *L. mucronata*, *M. crystallinum*, *M. nodiflorum*, *R. pictus*, and *S. glaucus* (Table S1 and Figure 3a). Therefore, these species were selected to study the effect of *C. polygonoides* on their physiological behavior.

Calculations of RII, based on the plant biomass, revealed that *M. nodiflorum*, *L. mucronata*, *M. crystallinum*, *S. glaucus*, and *R. pictus* received facilitation, while, *B. diandrus*, *C. maritima*, and *E. laciniatum* were inhibited under *C. polygonoides* (Figure 3b).

### 3.2. Effect of C. polygonoides Shrub on the Environmental Conditions of the Microhabitat

The measurements of the different environmental variables revealed that *C. polygonoides* reduced the temperature and PPFD under canopy compared to the outside (Table 1). The temperature under canopy decreased by 10.54% and 7.85% for the old and young shrubs, respectively, while the light intensity was lower by 95.11% and 89.08% for the old and young shrubs, respectively. In addition, the soil moisture content under canopy was significantly higher by 509.52% and 85.71% for old and young shrubs, respectively, compared with that outside canopy (Table 1). Moreover, soil organic carbon, total nitrogen, and salinity under canopy showed significant variations from those of the soil outside canopy. In this regard, the organic carbon content increased under the canopy by 67.92% and 47.80% for the old and young shrubs, respectively, while salinity content was reduced by 47.62% and 23.81% for the old and young shrubs, respectively (Table 1).

However, no significant variation was observed in the soil pH values between under- and outside canopy neither for old nor young shrubs. On the other hand, significant variations were detected between old and young shrubs regarding soil moisture and salinity, but not for organic carbon, total nitrogen, or pH (Table 1).

### 3.3. Effects on the Physiological Responses of Understory Plant Species

Except for *S. glaucus*, the plants under canopy showed significantly higher contents of *Chl* a than that of the counterparts collected from the outside canopies (Table 2). The same pattern was observed for *Chl* b contents, except for *M. nodiflorum*, which showed higher *Chl* b contents in plants growing outside the canopy (Table 2).

*Chl* a/b ratio in *S. glaucus*, *B. diandrus*, and *C. maritima* decreased in plants grown under canopy compared to those of the gap locations. However, this ratio increased for other understory species for plants under canopies. In a similar pattern, *M. nodiflorum* and *S. glaucus* plants under canopy showed more carotenoid contents (Table 2).

**Figure 4:**
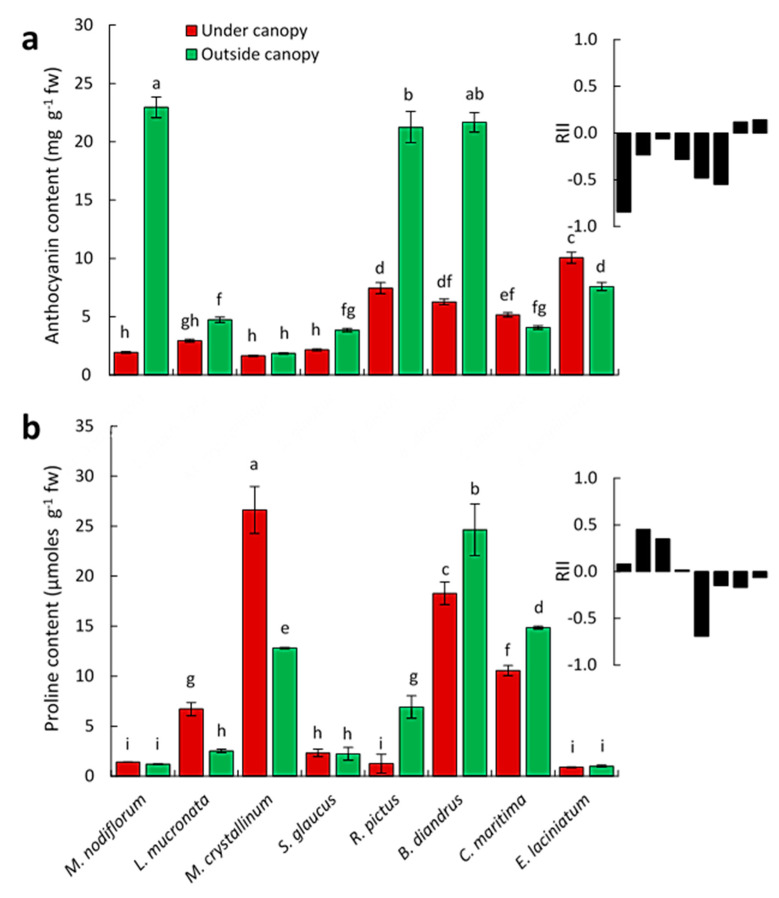
Effect of *C. polygonoides* shrubs on (**a**) anthocyanin content in the eight selected associated species under and outside. (**b**) proline content. Columns with the same letter are not significantly different after Duncan’s post-hoc test (*p* ≤ 0.05). Values are average ± standard error (*n* = 5). The relative interaction index (RII) was presented on the right as black columns.

Measurements of anthocyanin contents confirmed the field observation in that the facilitated plants (under canopy) were less reddish than counterparts. Except for *C. maritima* and *E. laciniatum*, the studied understory plants (under canopy) showed higher anthocyanin contents than that growing outside the canopy, while, no significant difference was observed in the anthocyanin content in case of *M. crystallinum* compared to the outside canopy plants (Figure 4a).

Moreover, the proline content was found to be significantly lower in *R. pictus*, *B. diandrus*, and *C. maritima* growing under canopy than that outside canopy. In contrast, *L. mucronata* and *M. crystallinum* showed higher proline contents in under- than outside canopy plants. For other plants, there were no significant differences, in proline content, between plants under- and outside canopy (Figure 4b).

**Figure 5:**
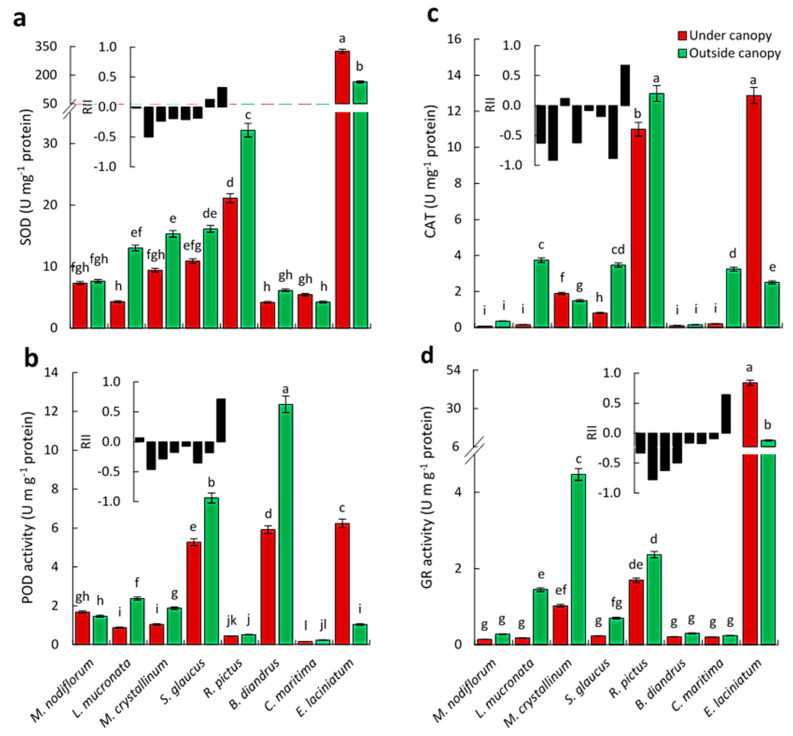
Antioxidant enzymes activity of the eight selected associated species in response to *C. polygonoides* shrubs (under and outside canopy) as well as the relative interaction index (RII) as black columns. (**a**) Superoxide dismutase (SOD) activity. (**b**) Peroxidase (POD) activity. (**c**) Catalase (CAT). (**d**) Glutathione reductases (GR) activity. Columns with the same letter are not significantly different after Duncan’s post-hoc test (*p* ≤ 0.05). Values are average ± standard error (*n* = 5).

The activity of SOD was significantly higher in *L. mucronata* and *M. crystallinum* and *R. pictus* plants growing outside the canopy than the under canopy plants. However, *E. laciniatum* plants growing outside canopy showed lower SOD activities compared with those under the canopy. The other species showed no significant differences in this concern (Figure 5a).

In contrast to *L. mucronata*, *M. crystallinum*, *S. glaucus*, and *B. diandrus*, the POD activity was significantly lower in *E. laciniatum* plants collected from places outside the canopy than the under canopy plants, while, no significant difference in the activity of this enzyme was detected between the other studied plants, under- and outside canopy Figure 5b.

For *L. mucronata*, *S. glaucus*, *R. pictus*, and *C. maritima* plants, the CAT activity was found to be significantly higher in plants growing outside than that under canopy, in contrast to *E. laciniatum* plants. For the other studied species, there were no significant differences observed in the activity of this enzyme between samples collected from outside and under canopy (Figure 5c). In contrast to *E. laciniatum*, the GR activity of *L. mucronata* and *M. crystallinum* was significantly higher in the plants growing outside than under canopy, while, no significant differences were detected for the rest of species with this regard (Figure 5d).

### 3.4. Mycorrhizal Fungi Associated with C. polygonoides Roots

**Figure 6:**
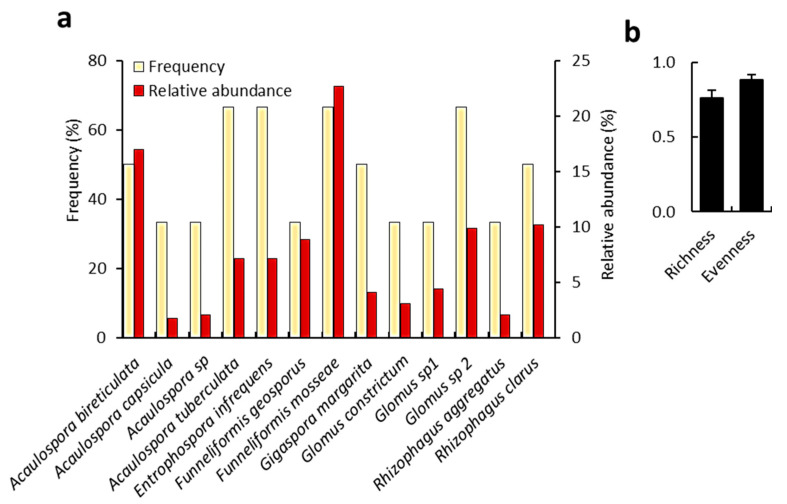
Diversity analyses of mycorrhizal fungi in the rhizosphere soil of *C. polygonoides*. (**a**) frequency and relative abundance. (**b**) Species diversity indexes (richness and evenness).

A total of 13 species and six genera of mycorrhizal fungi were identified in the rhizospheric soil of *C. polygonoides* from the selected sampling sites. The identified genera included *Acaulospora*, *Entrophospora*, *Funneliformis*, *Gigaspora*, *Glomus*, and *Rhizophagus*. Frequency and relative abundance of the identified fungi were are presented in Figure 6a. Among the observed fungi, *F. mosseae* was the most abundant (22.7%), while, the highest frequency was recorded for *A. tuberculata*, *E. infrequens*, *F. mosseae*, and *Glomus* sp. 2 (66.67% for each). Spore diversity (richness and evenness) of the identified mycorrhizal fungi are illustrated in Figure 6b. The average species richness was 0.76, while the species evenness was 0.88. The spore density in the studied sites ranged between 8.5 and 32.5 spores per 50 g of soil.

Mycorrhizal colonization levels in roots of the selected plants growing under and outside canopy are shown in Table 3. Of the eight evaluated plants, four species (*B. diandrus*, *E. laciniatum*, *L. mucronata,* and *S. glaucus*) were found to be colonized, to varying degrees, with mycorrhizal fungi. Although the frequencies of mycorrhization in these colonized roots were found to have moderate to high levels, the intensities of their mycorrhization were found in low levels. In contrast, the other four plant species (*M. crystallinum*, *M. nodiflorum*, *R. pictus,* and *C. maritima*) showed no mycorrhizal colonization.

### 3.5. Chemical Composition of C. polygonoides Root Extract

Using GC-MS analysis, 31 compounds at varied existence ratios were determined in the root extract (Table 4 and Figure S2). Among them, four compounds were detected as major components, namely; pyrogallol (33.00%), palmitic acid (12.02%), acetic acid (7.66%), and 4H-Pyran-4-one, 2,3-dihydro-3,5-dihydroxy-6-methyl (5.46%). In addition, five compounds were detected as minor components including; 3-methylpentane (4.46%), acetasol (3.93%), catechol (3.43%), *β*-hydroxyethyl isopropyl ether (2.48%), and citronellyl acetone (2.06%), while, the others were found as trace constituents.

## 4. Discussion

Results obtained in this study demonstrated that *C. polygonoides* shrubs provided either facilitation or inhibition to their associated plant species. The old shrubs provided facilitation for 18 and inhibited another 18 understory plant species, while the young shrubs provided facilitation to 13 and inhibited 9 plant species. It is clear that most of the typical halophytes were inhibited by *C. polygonoides* shrubs. These halophytes included perennial halophytes such as *Zygophyllum album*, *Z. aegyptium*, *Arthrocnemum macrostachyum*, as well as halophytic herbs and grasses such as *Bassia muricata*, *Parapholis incurva*, *Lolium perenne*, and *Paspalidium geminatum*. This inhibition can be ascribed to the competition for the resources as these species are well adapted for this stressful environment. On the other hand, the majority of the understory plant species received facilitation from *C. polygonoides* shrubs, however, the provided facilitation was species-specific dependant. The balance between facilitation and competition phenomena seems to vary based on life stage, physiology of the interacting species, intensity of the abiotic stress, and other biotic stresses such as allelopathy [39]. In arid and semiarid ecosystems, the net effects of the woody plants on their understory species usually shift between facilitation and interference depending on environmental conditions [40,41]. Under shrubs such as *C. polygonoides*, the light intensity is significantly reduced compared to open gaps and this makes a difference in water relations and temperature [2]. *C. polygonoides* is known for its ability to colonize deserts and grow in harsh conditions such as Mediterranean ecosystems marked by lengthy warm summers with elevated irradiance and little or scarce precipitation [42,43]. Therefore, we can say that the facilitation or inhibition of *C. polygonoides* shrubs is species dependent. The communities associated with *C. polygonoides* shrubs revealed facilitation regarding evenness than richness.

The mechanism of facilitation could be indirect biotic, abiotic via nutrient enrichment, or abiotic facilitation via microclimate amelioration [44]. In general, the young *C. polygonoides* shrubs showed more facilitation to the understory species than the old one, where the overall communities under the young *C. polygonoides* shrubs showed more richness as well as evenness. This can be attributed to the development of the shrub architecture as the branches of the old shrubs became very condensed by the time. This condensation may negatively affect the establishment of the other associated species. On the other hand, the old shrubs produce a condensed layer of plant litter which hinders the seeds from reaching the soil for establishment [9,45].

Based on biomass production, *M. nodiflorum*, *L. mucronata*, *M. crystallinum*, *S. glaucus*, and *R. pictus* received facilitation from the *C. polygonoides* shrubs. The aboveground biomass of *M. crystallinum* was reported to be 4-fold higher under the influence of *Eulychnia acida* in the Atacama Desert [46], and this facilitation was explained by direct and/or indirect positive effects. In agreement with the results obtained in this study, the *E. acida* retained more water and nutrients which provided more benefits to *M. crystallinum* [46]. *Mesembryanthemum crystallinum* performs as C_3_ plant in mesic environments but switched to CAM metabolism under drought stress. However, *M. crystallinum* performance becomes better in less saline habitat compared to salt marsh habitat along the Mediterranean coast of Egypt [20]. This may explain the facilitation received by *M. crystallinum* under *C. polygonoides* shrubs.

Compared to the gaps (outside canopy), the observed relatively high content of moisture beneath the canopy of *C. polygonoides* shrubs is in harmony with previous studies [41,47,48]. The canopy of *C. polygonoides* provided a shade that reduced the solar radiation and soil temperature for the understory plants and led to decline of PPFD and water evaporation, which may contribute to the high moisture content under the shrubs [49,50]. In addition, the dense vegetative growth allows the tree to condense fog water. On the other hand, the increment of the organic matter content under the canopy may indirectly contribute to the increased moisture content as it retains more water [51]. The understory species received a benefit from the water that is retained under canopy particularly in arid and semiarid ecosystems [19].

In addition, the reduced light intensity under canopy may protect the understory vegetation from the fatal photoinhibition and give the plants more benefits [52]. However, the heavy shading may hinder the photosynthesis as well [45], and this was clear in the present results as the young shrubs (low shading) provided more facilitation to the understory plant species than the old shrubs (high shading).

The salt concentration under the canopy in the present study may have led to a significant reduction in the plant diversity compared to the gaps (outside canopy) which played an important role in the facilitation for the understory species as the high salt concentrations can inhibit the plant growth due to the osmotic stress and ion toxicity [53]. The shrubs can mitigate the salinity stress via shading, which reduces the evaporation of the soil water under canopy as well as the condensation of water vapor, leading to maintaining the soil water potentials, which results in reduction of the soil salinity [2]. The study by Bertness and Yeh [54] reported that *Iva frutescens* shrub decreased soil salinity and facilitated the growth and survival of *Juncus gerardi* in salt marsh habitat due to the shade effect.

In dry ecosystems, the non-resource factors (temperature and light) become more important for the plant facilitation than the nutrients such as organic carbon and the total nitrogen [44]. In the present study, *C. polygonoides* shrubs significantly improved the soil nutrients such as organic C and total N. This observation has been reported for other shrubs in similar ecosystems such as *C. mongolicum*, *Nitraria sphaerocarpa*, *Haloxylon ammodendron* [50], *Retama sphaerocarpa* [55], and *Caragana intermedia* [56]. The higher soil contents of total N and organic C under old than young shrubs can be ascribed to the higher amount of litter under the old shrubs. Moreover, the facilitation effect of the shrubs provided more species richness for the understory species that indirectly increased the nutrient contents such as N and C [2]. The nutrients content has been reported to be positively correlated with the litter decomposition process, while the facilitation is negatively correlated with the phytotoxicity (allelopathy) of the litter materials [57].

The salt-tolerant plant species gained benefits such as mitigation of the soil salinity by the nurse plant. In contrast, the halophytic species were inhibited under the canopy of *C. polygonoides* shrubs, which can be ascribed to the competition rather than facilitation due to lower content of salinity. The light shapes the plant life through its influence on growth, pigment formation, and photosynthesis. The high contents of *Chl* a and *Chl* b of the understory plants compared to those growing outside canopy indicated that the extended vegetative development of the canopy decreased the underlying solar radiation and prevented the photoinhibition leading to conservation of the photosynthetic pigments of the understory plants. This result is in accordance with a previous study performed to evaluate the nursing effect of *Acacia auriculiformis* and *A. mangium* on *Castanopsis hystrix*, *Michelia macclurei*, and *Manglietia glauca* [8]. The high *Chl* b content of *M. nodiflorum* growing outside canopy may be due to the exposure to high ratio of red: far-red light compared to under canopy plants. Total chlorophyll of *M. nodiflorum* was reported to be higher in the plants subjected to high red: far-red light under greenhouse conditions [58].

The decrease in *Chl* a/b ratio for *S. glaucus*, *B. diandrus*, and *C. maritima* plants growing under canopy compared to those of the gap locations indicated the ability of these species to change the pattern of the pigment in response to their prevailing environments. This result is in agreement with that observed for *Castanopsis hystrix* under *Acacia* [8] to increase the amount of the light-harvesting complexes in antenna enriched with *Chl* b.

Carotenoids act as antioxidants in addition to their role in light collection and photoprotection. The observed reduction in carotenoids of *M. nodiflorum* under canopy is in accordance with the same result reported for *Castanopsis hystrix* which was facilitated by *Acacia* [8]. The opposite result was observed for the other species in the same study, as in the case of *Manglietia glauca*, indicating the photoinhibition facilitation ability of the overstory canopy.

Moreover, anthocyanins level was lower in the understory plants than in that growing outside canopy. The significantly lower level of anthocyanins in the understory species indicated another facilitation effect of *C. polygonoides* via alleviation of the radiation stress on these plants. Anthocyanins are produced in response to radiation stress and nutrient scarcity [59], and they are known for their ability to absorb UV radiation [60]. Supporting this result, the anthocyanins content in *Orbea variegata* was also reduced under the chenopod shrubs [61]. In addition, accumulation of anthocyanins may be linked to abiotic stress such as drought and salt stresses [62], which means that *C. polygonoides* provided a more suitable environment for the growth of the understory species compared to the prevailed stress conditions in the surrounding environment. However, *E. laciniatum* growing under the canopy of *C. polygonoides* in the present study showed more anthocyanins content compared to that growing outside canopy, this opposite behavior is unjustified, thereby further research is needed to understand this behavior.

Salt stress is usually accompanied by proline accumulation in plants [63]. In this study, the detected high level of proline in *R. pictus*, *B. diandrus*, and *C. maritima* plants growing outside canopy refers to the facilitative effect of *C. polygonoides* shrub on the same species growing under their canopy. However, the presence of *L. mucronata* and *M. crystallinum* under the canopy induced their proline level, an unexpected result that may be explained as a species-specific response to such environmental conditions. In stressed plants, the generated reactive oxygen species induce the antioxidant enzymes to scavenge these toxic compounds [64]. The reduced activities of SOD, POD, CAT, and GR in the understory plants indicated the ability of *C. polygonoides* to alleviate the oxidative stresses exerted on these plants.

The data obtained in this study showed a high diversity of mycorrhizal fungi in the rhizospheric soil of *C. polygonoides*. In this concern, a total of 13 species and 6 genera of mycorrhizal fungi were identified. In addition, mycorrhizal colonization was confirmed in roots of four of the eight selected plant species. In the mycorrhizal relationship, the plant provides the fungal partner with a carbon source from the photosynthesis process, while the latter enhances the plant growth, nutrients, and water uptake [65], and induces the plant resistance to biotic and abiotic stresses [66]. The presence of these diverse sets of mycorrhizal fungi in the rhizosphere of *C. polygonoides* demonstrated their probable contribution in the facilitative/inhibitive effect on the understory species through the formation of CMNs. This mycorrhization has the ability to mitigate the salinity stress in the plants [13]. This bio-amelioration of salinity stress is mediated with a diverse set of biochemical and physiological mechanisms including enhanced nutrient uptake, antioxidant metabolism, osmoprotection, regulating of ionic homeostasis, and maintenance of cell ultrastructure [67]. These probable mechanisms may explain the facilitation effect exerted on the understory plants in the present study. With regard to the non-mycorrhizal plant species in this study, absence of mycorrization in their roots is an expected result where they are members of well-known non-mycorrhizal plant families [68]. Moreover, arbuscular mycorrhizal fungi have important roles in reducing soil erosion, nutrient leaching, and enhancing soil texture and fertility [14]. In other words, they can differently change plant coexistence, induce plant productivity, and alter aboveground biodiversity-ecosystem relationships depending on the fungal and plant species involved in the CMN [69].

Gas chromatography-mass spectrometry analysis revealed the existence of 31 chemical compounds in the root extract of *C. polygonoides*. Among them, different bioactive compounds with well-known antimicrobial and/or allelopathic activities were detected. Pyrogallol and catechol are phenolic allelochemicals which are synthesized in plants via the shikimate pathway and have important roles as protective and antioxidant agents against attacking microbes and as signaling molecules in the plant-pathogen interaction [70]. Both bioactive compounds have been widely reported as antibacterial and antifungal agents against varied microorganisms [71]. Their antimicrobial mode of action refers mostly to their inhibitory effect on the enzymes by reacting with sulfhydryl groups or other non-protein interactions. Their microbial-toxicity depends on the position and number of the hydroxyl groups on their aromatic ring [72]. Soil microbial communities play a basic role in the facilitative effect of the nurse plant on the under canopy plants affecting their competition, diversity, fitness, and nutrient availability [73,74].

Nurse plants influence the occurrence, distribution, viability, diversity of the soil microbiota, and their interactions with the beneficiary plant species [74]. Moreover, recent researches have reported the role of the nurse plant in changing the composition of the soil microbiome by enhancing the beneficial microorganisms involved in nutrient mineralization and plant growth regulation and suppressing the harmful microorganisms through secretion of root exudates [75]. In this regard, it was found that flavonoids secreted as plant exudates act as chemical attractants to *Rhizobia* [76]. Moreover, 4H-Pyran-4-one, 2,3-dihydro-3,5-dihydroxy-6-methyl, which is determined as a major flavonoid compound in the present study, has been reported as an antifungal agent [77]. Indeed, root exudates act as chemical messengers to regulate the microbial chemotaxis and interactions via a diverse array of molecular networks [78]. Functions of root exudates include control of the plant-microbe association, promotion for beneficial symbiotic microorganisms, protection against herbivores, suppression of other competent plants, and pathogenic microorganisms, as well as improvement of the chemo-physical soil properties [79].

On the other hand, pyrogallol and catechol (major compounds in *C. polygonoides* root extract) are considered as precursors in the synthesis of humic-like polymers via the so-called humification process [80]. In addition, phenolics secreted as root exudates once they are incorporated into the soil and decomposed by the microbial enzymatic action, they act as a potential geoengineering tool regulating soil organic matter decomposition, N availability, nutrient cycling, and humus formation which affect soil structure and fertility [81]. Moreover, the toxicity of the allelochemicals in the soil is reduced with time due to microbial degradation, not only this but may be used as nutrients by microbes. This biochemical dynamics of the allelochemicals can potentially affect key ecosystem processes, including plant growth and microbial activity and thereby affect the facilitation process of the nurse plants [57].

## 5. Conclusions

The nurse plant, *C. polygonoides,* along the Mediterranean coast of Egypt provides either facilitation or suppression for the understory species in a species-specific manner. In addition, the shrub affects the microhabitats by altering light intensity, soil temperature, soil composition, particularly the soil moisture, salinity, and organic matter content. The present study revealed high diversity and evenness of mycorrhizal fungi in the rhizosphere soil of *C. polygonoides* that seem to play significant and crucial roles and may explain their facilitative/inhibitive effect on the neighboring understory plants via CMNs. This facilitated effect was reflected in the enhanced growth performance, adaptation, and stress tolerance of the understory species. On the other hand, the present study showed that the roots of *C. polygonoides* have different bioactive compounds with antimicrobial and/or allelopathic activities such as pyrogallol and catechol. These compounds have an inhibitive effect on the pathogens in the soil microbiome, providing protection for the nurse plant and their understory species. Moreover, these bioactive compounds, as allelochemicals, may be responsible for the suppression of the understory species. However, the allelochemicals act also as stimulatory compounds, particularly at a lower concentration.

It became evident that the facilitation of the understory species is not limited to a single mechanism, but obviously includes a set of interrelated mechanisms. Moreover, the varied responses of the studied understory species revealed that the response to facilitation provided by *C. polygonoides* is species-specific. Based on the data obtained in the present study, it can be concluded that the *C. polygonoides* has an implication for the restoration of the endangered ecosystem along the Mediterranean coast in Egypt, and maybe in other coastal areas in the world with similar climate and conditions, which may improve the natural vegetation establishment. A further study is recommended for the evaluation of *C. polygonoides* as a nurse plant via a long-term field experiment, maybe on a global scale, with the implication of mechanisms of either facilitation or inhibition on a large set of understory species.

## Figures and Tables

**Table 1:** Photosynthetic photon flux density (PPFD) and soil parameters under the canopy of *C. polygonoides* and outside the canopy. Values are average ± standard error (*n* = 15). Means followed by different letters within each row are significantly different after Duncan’s post-hoc test.

Parameter	Outside Canopy	Under Canopy		F-Value
		Old	Young	
PPFD (μmol m^−1^ s^−1^)	1353.85 ± 58.79 ^a^	66.20 ± 7.65 ^b^	147.90 ± 17.13 ^b^	174.68 ***
Temp. (°C)	29.87 ± 0.70 ^a^	26.72 ± 0.28 ^b^	27.53 ± 0.60 ^b^	5.72 *
Moisture content (%)	0.21 ± 0.01 ^c^	1.28 ± 0.05 ^a^	0.39 ± 0.06 ^b^	1726.17 ***
Organic carbon (g kg^−1^)	1.59 ± 0.15 ^b^	2.67 ± 0.34 ^a^	2.35 ± 0.40 ^a^	6.26 **
Total nitrogen (g kg^−1^)	8.75 ± 0.18 ^b^	9.52 ± 0.28 ^a^	9.88 ± 0.46 ^a^	5.95 **
pH	8.52 ± 0.12 ^a^	8.53 ± 0.10 ^a^	8.36 ± 0.14 ^a^	0.91 ^ns^
EC (mS cm^−1^)	0.21 ± 0.01 ^a^	0.11 ± 0.02 ^b^	0.16 ± 0.03 ^a^	7.30 **

Different superscript letters within each row means values significant difference after Duncan’s post-hoc test, * *p* < 0.05, ** *p* < 0.01, *** *p* < 0.001, ns: non-significant.

**Table 2:** Water content and photosynthetic pigments of the eight selected associated under canopy and outside canopy of *C. polygonoides* as well as the relative interaction index (RII).

Plant Species		Water Content (%)	Chl a (µg g^−1^ fw)	Chl b (µg g^−1^ fw)	Total Chls (µg g^−1^ fw)	Chl a/Chl b	Carotenoids (µg g^−1^ fw)
*Mesembryanthemum nodiflorum*	Under	5.55 ± 1.70 ^E^	84.53 ± 2.57 ^G^	102.88 ± 0.80 ^C^	187.41 ± 3.20 ^CD^	0.82 ± 0.02 ^I^	10.16 ± 0.08 ^D^
	Outside	3.62 ± 0.37 ^F^	70.72 ± 0.55 ^I^	111.01 ± 0.87 ^A^	181.73 ± 1.42 ^E^	0.64 ± 0.00 ^K^	12.38 ± 0.10 ^B^
	***RII***	0.21	0.09	−0.04	0.02	0.13	−0.10
*Launaea mucronata*	Under	5.44 ± 0.18 ^E^	48.71 ± 1.48 ^J^	15.92 ± 0.12 ^N^	64.64 ± 1.57 ^I^	3.06 ± 0.08 ^B^	3.18 ± 0.02 ^L^
	Outside	6.27 ± 0.08 ^D^	21.72 ± 0.17 ^L^	12.95 ± 0.10 ^O^	34.67 ± 0.27 ^L^	1.68 ± 0.00 ^H^	2.66 ± 0.02 ^N^
	***RII***	−0.07	0.38	0.10	0.30	0.29	0.09
*M. crystallinum*	Under	12.25 ± 0.07 ^A^	79.77 ± 2.42 ^H^	109.85 ± 0.86 ^B^	189.62 ± 3.11 ^C^	0.73 ± 0.02 ^J^	7.46 ± 0.06 ^G^
	Outside	8.10 ± 0.07 ^B^	44.60 ± 0.35 ^J^	77.85 ± 0.61 ^E^	122.45 ± 0.95 ^H^	0.57 ± 0.00 ^L^	5.29 ± 0.04 ^K^
	***RII***	0.20	0.28	0.17	0.22	0.12	0.17
*Senecio glaucus*	Under	2.70 ± 0.67 ^G^	36.96 ± 1.12 ^K^	18.30 ± 0.14 ^M^	55.26 ± 1.23 ^J^	2.02 ± 0.05 ^G^	2.91 ± 0.02 ^M^
	Outside	2.85 ± 0.51 ^G^	33.82 ± 0.026 ^K^	15.77 ± 0.12 ^N^	49.58 ± 0.39 ^K^	2.14 ± 0.00 ^F^	3.09 ± 0.02 ^L^
	***RII***	−0.03	0.04	0.07	0.05	−0.03	−0.03
*Rumex pictus*	Under	7.46 ± 0.58 ^C^	86.82 ± 2.64 ^G^	38.32 ± 0.30 ^K^	125.14 ± 2.86 ^H^	2.27 ± 0.06 ^E^	6.82 ± 0.05 ^I^
	Outside	2.40 ± 0.48 ^H^	16.41 ± 0.13 ^M^	21.02 ± 0.16 ^L^	37.43 ± 0.29 ^I^	0.78 ± 0.00 ^IJ^	2.90 ± 0.02 ^M^
	***RII***	0.51	0.68	0.29	0.54	0.49	0.40
*Bromus diandrus*	Under	2.50 ± 0.02 ^H^	242.63 ± 7.37 ^A^	87.38 ± 0.68 ^D^	330.01 ± 7.88 ^A^	2.78 ± 0.07 ^C^	14.21 ± 0.11 ^A^
	Outside	1.41 ± 0.02 ^J^	139.24 ± 1.09 ^C^	42.98 ± 0.33 ^I^	182.22 ± 1.42 ^DE^	3.24 ± 0.00 ^A^	7.30 ± 0.06 ^H^
	***RII***	0.28	0.27	0.34	0.29	−0.08	0.32
*Cakile maritima*	Under	2.84 ± 0.04 ^G^	128.81 ± 3.91 ^D^	57.12 ± 0.45 ^G^	185.93 ± 4.25 ^CDE^	2.25 ± 0.06 ^D^	7.95 ± 0.06 ^E^
	Outside	2.86 ± 0.09 ^G^	109.33 ± 0.85 ^E^	46.14 ± 0.36 ^H^	155.47 ± 1.21 ^F^	2.37 ± 0.00 ^E^	7.65 ± 0.06 ^F^
	***RII***	0.00	0.08	0.11	0.09	−0.02	0.02
*Erodium laciniatum*	Under	3.60 ± 0.01 ^F^	175.70 ± 5.34 ^B^	75.89 ± 0.59 ^F^	251.59 ± 5.78 ^B^	2.32 ± 0.06 ^D^	11.47 ± 0.09 ^C^
	Outside	2.08 ± 0.00 ^I^	97.75 ± 0.76 ^F^	41.68 ± 0.32 ^J^	139.43 ± 1.09 ^G^	2.35 ± 0.00 ^DE^	6.63 ± 0.05 ^J^
	***RII***	0.27	0.29	0.29	0.29	−0.01	0.27
F-value		3129.25	1427.61	14651.38	2127.02	1545.38	10693.12

Values with the same letter within each column are not significantly different after Duncan’s post-hoc test (*p* ≤ 0.05). Values are average ± standard error (*n* = 5).

**Table 3:** Mycorrhizal colonization level (%) in roots of the selected plant species under and outside the canopy of *C. polygonoides*.

Plant Species	Under Canopy			Outside Canopy		
	F	M	A	F	M	A
*Bromus diandrus*	95 *	2.35	0	75	2.15	0.17
*Cakile maritima*	0	0	0	0	0	0
*Erodium laciniatum*	65	2.65	0	60	1.67	0
*Launaea mucronata*	30	0.3	0	30	0.3	0
*Mesembryanthemum crystallinum*	0	0	0	0	0	0
*M. Nodiflorum*	0	0	0	0	0	0
*Rumex pictus*	0	0	0	0	0	0
*Senecio glaucus*	91	6.82	1.5	26.7	0.53	0.03

* Each value represents the mean of 40 root segments of each plant species, F = frequency of root colonization, M = intensity of cortical colonization, and A = frequency of arbuscules.

**Table 4:** Chemical composition of *C. polygonoides* root extract as detected by GC-MS system.

Peak	Rt *	Conc. (%)	Compound Name
1	3.490	1.30	1,2-Dichloro-tetramethyldisilane
2	3.855	1.30	4-Phenylglutamic acid
3	5.980	1.04	5-(2-Aminopropyl)-2-methylphenol
4	6.079	12.02	Palmitic acid
5	6.170	1.16	1,6-Dideoxy-2,4-O-methylenehexitol
6	6.379	2.48	*β*-hydroxyethyl isopropyl ether
7	6.810	4.46	3-methylpentane
8	7.475	0.02	Malonodinitrile, 2-(2,4,6-trichlorophenylhydrazono)
9	8.372	7.66	Acetic acid
10	8.510	3.93	Acetasol
11	11.504	1.07	1,2,4-Butanetriol
12	13.975	1.04	3-[4-(2-Methoxyphenyl)-1-piperazinyl]-1-propanol
13	14.940	5.46	4H-Pyran-4-one, 2,3-dihydro-3,5-dihydroxy-6-methyl
14	15.386	3.43	Catechol
15	18.108	33.00	Pyrogallol
16	18.265	2.06	Citronellylacetone
17	18.300	1.05	2-Chloro-2-propenyl 4-methylphenyl ether
18	18.330	1.29	3-Methoxy-5-methyl-4-nitrophthalic acid
19	18.370	1.09	6-Acetoxy-4-methyl-hept-4-enoic acid
20	18.435	1.39	Methyl 6-methyl-2-oxo-2H-pyran-5-carboxylate
21	18.672	1.25	Cyclohexane-1,2-dimethanol, diacetate
22	18.918	1.03	1-(3,6-Dimethyl-2-pyrazinyl)-1-propanone
23	21.101	1.36	5,5′-Diamino-3,3′-bis-1,2,4-triazole
24	22.095	1.11	5-Bromouridine
25	22.227	1.26	1,4-Cyclooctanediol
26	23.228	1.23	1-Methylverbenol
27	25.758	1.33	4-Nitrophenyl hexopyranoside
28	25.811	1.16	1H-Pyrrole-2,5-dione, 3-.beta.-D-ribofuranosyl
29	27.380	1.09	4-Nitrophenyl hexopyranoside
30	28.921	1.91	1-Ethyloctyl methyl sulfide
31	31.490	1.02	Trimethylsilyl 12-oxooctadecanoate

* Rt: retention time (min).

## Supplementary Materials

The following are available online at https://www.mdpi.com/2079-7737/9/8/232/s1, Figure S1: Measurments of C. polygonoides shrubs measurements within the studied locations in the study area, Figure S2: GC-MS chromatograms of the C. polygonoides root extract, Table S1: The plant species composition, families, life forms, chorotypes, and frequency percentage of the recorded species associated with C. polygonoides shrubs.
