# Supplementary material for: Calligonum polygonoides L. Shrubs Provide Species-Specific Facilitation for the Understory Plants in Coastal Ecosystem
Source: Biology (Basel). 2020 Aug 17;9(8):232. doi: 10.3390/biology9080232 (PMC7464817; doi:10.3390/biology9080232)
Supplement: Supplementary file 1 [file biology-09-00232-s001.pdf]

# *Calligonum polygonoides* L. Shrubs Provide Species-Specific Facilitation for the Understory Plants in Coastal Ecosystem

**Ahmed M. Abd-ElGawad[1,2*±], Younes M. Rashad[3], Ahmed M. Abdel-Azeem[4], Sami A. Al-Barati[5], Abdulaziz M. Assaeed[1] and Amr M. Mowafy[2±]**

## Supplementary Materials

**Table S1.** The plant species composition, families, life forms, chorotypes, and frequency (F) percentage of the recorded species associated with *C. polygonoides* shrubs.

| No. | Plant species | Family | Life | Chorotype | F (%) |
|---|---|---|---|---|---|
| *Perennials* | | | | | |
| 1 | *Alhagi graecorum* Boiss. | Fabaceae | H | PAL | 3.70 |
| 2 | *Arthrocnemum macrostachyum* (Moric.) K. | Chenopodiaceae | Ch | ME+SA-SI | 11.11 |
| 3 | *Cynanchum acutum* L. | Ascliapedicaeae | H | ME+IR-TR | 3.70 |
| 4 | *Cynodon dactylon* (L.) Pers. | Poaceae | G | PAN | 7.41 |
| 5 | *Cyperus capitatus* Vand. | Cyperaceae | G | ME | 3.70 |
| 6 | *Echinops spinosus* L. | Asteraceae | H | ME+SA-SI | 3.70 |
| 7 | *Launaea mucronata* (Forssk.) Muschl. | Asteraceae | H | ME+SA-SI | 70.37 |
| 8 | *Pancratium maritimum* L. | Amaryllidaceae | G | ME | 11.11 |
| 9 | *Paspalidium geminatum* (Forssk.) Stapf. | Poaceae | He | PAL | 3.70 |
| 10 | *Phragmites australis* (Cav.) Trin ex Steud. | Poaceae | G, He | COSM | 3.70 |
| 11 | *Reicharadia tingitana* (L.) Roth | Asteraceae | H | ME+SA-SI | 14.81 |
| 12 | *Silene succulenta* Forssk. | Caryophyllaceae | H | ME | 3.70 |
| 13 | *Suaeda pruinosa* Lange | Chenopodiaceae | Ch | ME | 3.70 |
| 14 | *Zygophyllum aegyptium* Hosny | Zygophyllaceae | Ch | ME | 11.11 |
| 15 | *Zygophyllum album* L. | Zygophyllaceae | Ch | ME+SA-SI | 7.41 |
| *Annuals* | | | | | |
| 16 | *Aegilops bicornis* (Forssk.) Jaub & Spach | Poaceae | Th | ME+SA-SI | 7.41 |
| 17 | *Anchusa humilis* (Desf.) I M. Johnst. | Boraginaceae | Th | ME+SA-SI | 3.70 |
| 18 | *Bassia indica* (Wight) A. J. Scott | Chenopodiaceae | Th | S-Z+IR-TR | 18.52 |
| 19 | *Bassia muricata* (L.) Asch. | Chenopodiaceae | Th | SA-SI+IR-TR | 3.70 |
| 20 | *Brassica tournefortii* Gouan | Brassicaceae | Th | ME+IR-TR+SA-SI | 18.52 |
| 21 | *Bromus diandrus* Roth. | Poaceae | Th | ME | 48.15 |
| 22 | *Cakile maritima* Scop. | Brassicaceae | Th | ME+ER-SR | 66.67 |
| 23 | *Carduus pycnocephalus* L. | Asteraceae | Th | ME+IR-TR | 25.93 |
| 24 | *Carthamus tenuis* (Boiss. & Blanche) Bornm. | Asteraceae | Th | ME | 14.81 |
| 25 | *Chenopodium murale* L. | Chenopodiaceae | Th | COSM | 11.11 |
| 26 | *Cutandia maritima* (L.) Barbey | Poaceae | Th | ME+IR-TR+SA-SI | 22.22 |
| 27 | *Emex spinosa* (L.) Campd. | Polygonaceae | Th | ME+SA-SI | 3.70 |
| 28 | *Erodium laciniatum* (Cav.) Willd. | Geraniaceae | Th | ME | 22.22 |
| 29 | *Hordeum murinum* L. | Poaceae | Th | ME+IR-TR+ER-SR | 14.81 |
| 30 | *Ifloga spicata* (Forssk.) Sch. Bip. | Asteraceae | Th | ME+SA-SI | 18.52 |
| 31 | *Lolium perenne* L. | Poaceae | Th | ER-SR+ME+IR-TR | 3.70 |
| 32 | *Malva parviflora* L. | Malvaceae | Th | ME+IR-TR | 14.81 |

| | | | | | |
|---|---|---|---|---|---|
| 33 | *Mesembryanthemum crystallinum* L. | Aizoaceae | Th | ME+ER-SR | 29.63 |
| 34 | *Mesembryanthemum nodiflorum* L. | Aizoaceae | Th | ME+SA-SI+ER-SR | 25.93 |
| 35 | *Parapholis incurva* (L.) C. E. Hubb. | Poaceae | Th | ME+IR-TR+ ER-SR | 3.70 |
| 36 | *Plantago squarrosa* Murray | Plantaginaceae | Th | ME | 3.70 |
| 37 | *Rumex pictus* L. | Polygonaceae | Th | ME+SA-SI | 77.78 |
| 38 | *Salsola kali* L. | Chenopodiaceae | Th | COSM | 18.52 |
| 39 | *Senecio glaucus* L. | Asteraceae | Th | ER-SR+ME+IR-TR | 62.96 |
| 40 | *Urospermum picroides* (L.) F. W. Schmidt | Asteraceae | Th | ME+IR-TR | 3.70 |

H: Hemicryptophytes, Ch: Chamaephytes, G: Geophytes, He: Helophytes, Th: Therophytes, PAL: Palaeotropical, ME: Mediterranean, SA-SI: Saharo-Sindian, IR-TR: Irano-Turanian, PAN: Pantropical, COSM: Cosmopolitan, ER-SR: Euro-Siberian.

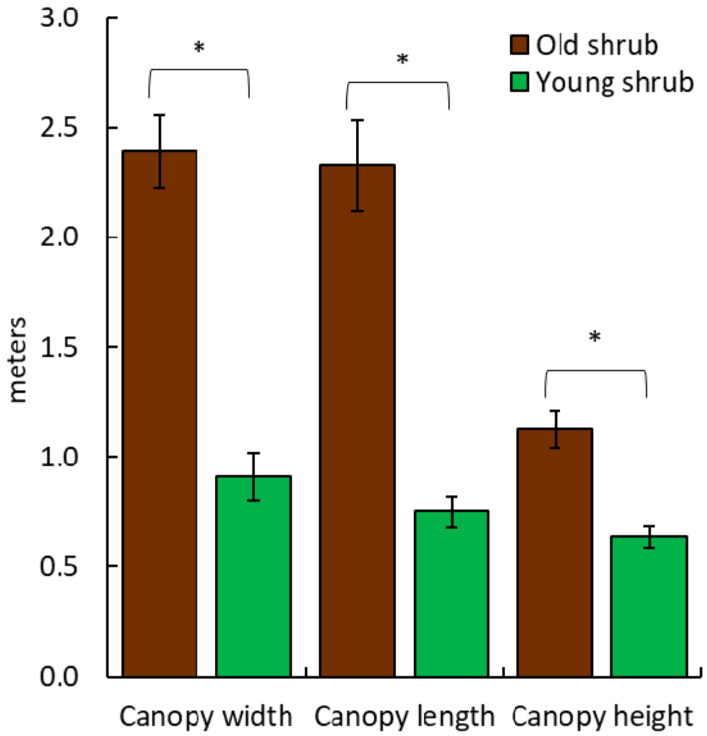


**Figure S1.** Measurments of *C. polygonoides* shrubs measurements within the studied locations in the study area. Values are average ± standard error ($n = 40$). * represents significant difference at $P < 0.05$ (two-tailed *t-test*).

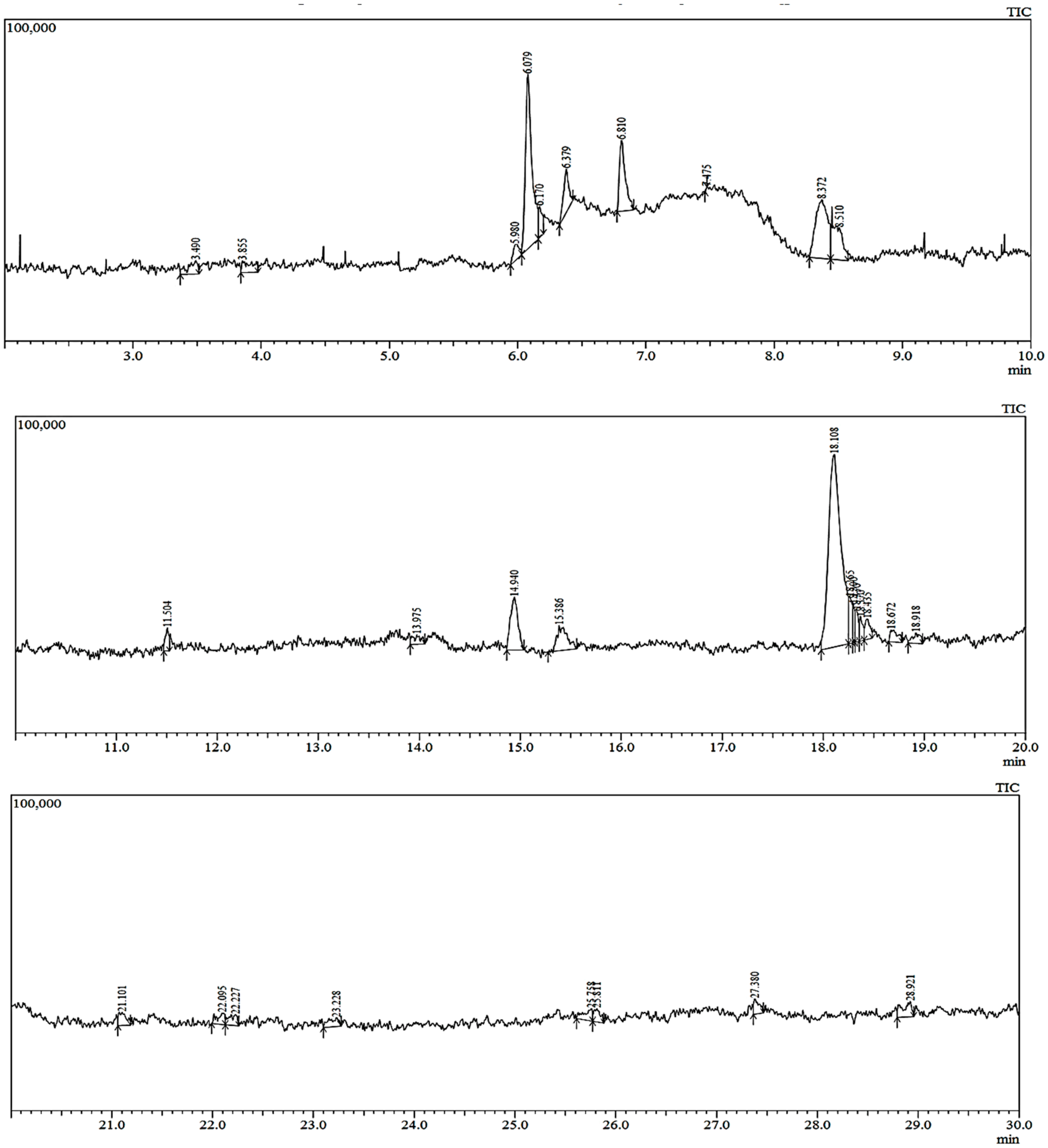


**Figure S2.** GC-MS chromatograms, in a sequence time, of the *Calligonum polygonoides* root extract. The peaks with a number showing the identified chemical compounds.
